# A Study of the Relationship Between Gestational Diabetes Mellitus and Antenatal Depression Using the Edinburgh Postnatal Depression Scale

**DOI:** 10.7759/cureus.102800

**Published:** 2026-02-01

**Authors:** Sunita Murmu, Rukhsar B Anwar Khan, Manoj Sahoo

**Affiliations:** 1 Obstetrics and Gynaecology, Tata Main Hospital, Jamshedpur, IND; 2 Psychiatry, Tata Main Hospital, Jamshedpur, IND

**Keywords:** antenatal care, antenatal depression, edinburgh postnatal depression scale (epds), gestational diabetes mellitus, pregnancy

## Abstract

Background: During pregnancy, a mother experiences a wide range of physiological as well as psychological changes. Maternal depression is one of the most common psychiatric disorders that occurs during pregnancy as well as after childbirth. If undiagnosed and untreated, it can lead to various maternal health issues, higher infant morbidity and mortality, and can also affect the growth, nutritional health, and cognitive, intellectual, and socioemotional development of the child. Women with diabetes during pregnancy are a high-risk group for developing mental health problems, and this has attracted worldwide attention. This study was therefore conducted to assess the relationship between gestational diabetes mellitus and antenatal depression and to identify factors associated with antenatal depression among the study participants.

Methods: The study included 480 pregnant women, of whom 240 had gestational diabetes mellitus (GDM) and 240 did not. The Edinburgh Postnatal Depression Scale (EPDS) was used to measure symptoms of depression in pregnant women attending routine antenatal care (ANC) visits. The scale was administered at 28 weeks of gestation and at the last antenatal visit prior to delivery. Descriptive statistics, logistic regression, and multiple regression analyses were performed to analyse the data.

Results: The final analysis was conducted on 467 study participants, of whom 232 pregnant women were diagnosed with GDM, whereas 235 pregnant women did not have GDM. The overall prevalence of significant depressive symptoms among the study participants was 11.6%. Thirty-seven (15.9%) pregnant women with GDM and 17 (7.2%) women without GDM had significant symptoms of antenatal depression (EPDS score ≥ 12). Pregnant women who exhibited significant symptoms of antenatal depression had a higher mean age (p = 0.039). Lower socioeconomic status (p = 0.038), limited social support (p = 0.046), and a sedentary lifestyle (p < 0.001) were associated with the presence of significant symptoms of antenatal depression.

Conclusions: The results suggest that symptoms of depression are more common in women with gestational diabetes. Therefore, assessment and education regarding this condition during the antenatal period are important.

## Introduction

Depression is an illness commonly characterized by persistent low mood, loss of interest in enjoyable activities, and reduced quality of life. As the perinatal period is accompanied by several hormonal changes, women during this time are more susceptible to developing depression [1]. Antenatal depression refers to depressive episodes occurring during pregnancy and is characterised by a range of emotional, cognitive, behavioural, and somatic symptoms. Common emotional symptoms include persistently low mood, anhedonia, feelings of hopelessness or excessive guilt, irritability, and anxiety. Cognitive symptoms include impaired concentration, indecisiveness, negative self-appraisal, and recurrent thoughts of death and self-harm. Behavioural symptoms may present as social withdrawal and reduced self-care. Somatic symptoms commonly reported include sleep disturbances, fatigue, appetite changes, and psychomotor agitation or retardation; however, symptoms require careful interpretation due to overlap with normal physiological changes of pregnancy. The clinical diagnosis of antenatal depression is based on the Diagnostic and Statistical Manual of Mental Disorders, Fifth Edition (DSM-5) criteria for major depressive disorder. DSM-5 requires the presence of at least five depressive symptoms persisting for a minimum of two weeks, with at least one symptom being either depressed mood or loss of interest or pleasure [2]. Globally, antenatal depression is found to affect about 15% to 65% of women during pregnancy [3]. It can negatively affect fetal growth and lead to poor outcomes, including preterm birth, low birth weight, impaired fetal brain development, and reduced coping ability in the child’s later life [4]. The Edinburgh Postnatal Depression Scale (EPDS) is a 10-item screening tool for postnatal depression and uses a Likert-type scale (0-3). Responses are scored as 0, 1, 2, and 3 based on the seriousness of the symptom. Items 3, 5 to 10 are reverse scored (i.e., 3, 2, 1, and 0). The total score is found by adding together the scores for each of the 10 items. The items are summed to give a total score, which can range from 0 to 30, with higher scores indicating greater symptoms of depression. The recommended cutoff score to indicate antenatal depression is 12/13, resulting in a sensitivity of 64% and specificity of 95%. It has also been validated for screening pregnant women for depression with evidence of acceptable reliability (α = 0.87) [5-7].

The World Health Organization (1999) report provides a fundamental definition, which states, “Gestational diabetes is a carbohydrate intolerance resulting in hyperglycemia of variable severity with onset or first recognition during pregnancy” [8]. In recent years, gestational diabetes mellitus (GDM) has emerged as a common condition during pregnancy. It has multiple adverse implications, including hypertension, polyhydramnios, and preterm labour in the mother, and fetal macrosomia, birth injury, respiratory distress, and hypoglycaemia in the infant [9,10]. There is a huge variation in the prevalence of GDM globally, depending on screening strategies, diagnostic criteria, and the background population’s ethnic composition [11]. India, being the most populous country in the world, has the prevalence of GDM ranging from 3 to 35% [12]. Currently, the Diabetes in Pregnancy Study Group of India (DIPSI) advocates for universal screening using a single non-fasting 75-gram oral glucose tolerance test, with a two-hour value of >140 mg/dL being diagnostic of GDM [13].

Depression and diabetes are frequently reported to coexist in the same pregnant women, which may be attributed to the common biological and behavioural mechanisms that underlie both depression and diabetes [14]. Increased inflammation, hyperactivity of the sympathetic nervous system and hypothalamic pituitary adrenal axis, low levels of physical activity, unhealthy dietary habits, low socioeconomic status, and chronic stress are some common factors supposed to be responsible for both conditions [15,16]. Pregnancy is a stressful life event and is often associated with anxiety and depression, which in many cases remain undetected and untreated. Moreover, GDM is also one of the most common complications of pregnancy. While some evidence from Western countries supports an association between gestational diabetes and depression [17], no studies of a similar nature have been conducted in the Indian pregnant population. Considering the potential negative consequences of GDM and antenatal depression, and the scarcity of information regarding these conditions in India, the present study was undertaken to determine whether pregnant women with gestational diabetes mellitus have a higher prevalence of antenatal depression compared to women without gestational diabetes mellitus, and to explore factors associated with antenatal depression among the study participants.

## Materials and methods

Study design and participants

The present study was a hospital-based case-control study conducted from August 2023 to July 2024 in the Department of Obstetrics and Gynaecology, Tata Main Hospital, Jamshedpur, India. All pregnant women attending the outpatient department at 28 weeks of gestation for a routine check-up constituted the source population of the study. The DIPSI method was used for screening and confirmatory testing of GDM. A sample size was calculated based on the formula:

$n=\frac{(z_{1-&alpha;/2} + z_{&beta;})^2 [ p_{1} (1-p_{1})+p_{2} (1-p_{2})]}{(p_{1}-p_{2})^2}$

where Z_1-α/2_ = 1.96 (level of confidence is considered as 95%), Z_𝛽_ = 0.84 (80% power of the study is assumed), p_1_ = 0.20, and p_2_ = 0.13. So, for 95% confidence level and 80% power, the minimum estimated sample size was 437. After adding 10% allowance for loss to follow up, the final sample size calculated was 480. The cases were pregnant women who were diagnosed with GDM; controls were pregnant women who did not have GDM.

Inclusion criteria

Pregnant women 18 years or older attending the outpatient department for a routine antenatal visit at 28 weeks of gestation were included.

Exclusion criteria

Pregnant women with uncertain gestational age, pre-existing diabetes mellitus (either type 1 or type 2), and associated high-risk condition (e.g., gestational hypertension, placenta praevia) were excluded.

Eligible cases and controls who met the inclusion criteria were recruited consecutively until the desired sample size was achieved. Controls were selected in a 1:1 ratio with cases to ensure comparability between the two groups. 

Methodology

Informed written consent was obtained after explaining the purpose of the study and assuring participants of the anonymity and confidentiality of the data provided. A detailed history was elicited from the patients, including sociodemographic parameters such as age, family type, education, socioeconomic status, and perceived social support from family, peers, and society. Reproductive and obstetric parameters, including gravida, history of pregnancy loss, complications in previous pregnancy, and the intent of the current pregnancy, were noted. Pre-pregnancy body mass index and lifestyle factors were recorded. A history of past depression that required counselling or the use of medications was self-reported by the participants. EPDS, a 10-item tool, was used to measure the symptoms of depression in pregnancy. Each item was scored on a Likert-type scale (0-3), and the scores were then summed. The scale was administered at 28 weeks of gestation and during the last antenatal visit prior to delivery. Based on the individual item scores, the total score was calculated at both visits. A cutoff score of greater than or equal to 12 was used to classify women as having significant symptoms of depression, and the participants were referred to a psychiatrist for proper diagnosis and management.

Statistical analysis

The collected data were organized and tabulated in Microsoft Excel 2016 (Microsoft Corporation, Redmond, Washington), and statistical analysis was done using IBM SPSS Statistics for Windows, Version 23 (Released 2015; IBM Corp., Armonk, New York). All continuous variables were expressed as mean ± standard deviation (SD), and categorical variables were expressed as relative frequency and percentage. Student's t-test was used to compare two independent groups of normally distributed continuous variables. The Mann-Whitney U test was used to compare two independent groups of skewed (non-parametric) continuous variables. The chi-square test, with or without Yates' correction, was used to compare categorical variables. Fisher’s Exact test was used for categorical data when at least one expected cell value was less than 5. A p-value < 0.05 was considered statistically significant.

Ethical approval

The protocol of the research study was submitted to the Institutional Ethics Committee of Tata Main Hospital, Jamshedpur, India, and the study was initiated after obtaining their approval (vide IEC reference no. TMH/IEC/JUNE/029/22 dated June 4, 2022).

## Results

The final analysis was conducted on 467 participants, of whom 232 pregnant women were diagnosed with GDM (cases) and 235 did not have GDM (controls). The overall prevalence of significant depressive symptoms among the study participants was 11.6%. The majority of the participants were aged between 26 and 30 years, comprising 112 (48.3%) cases and 107 (45.5%) controls (Table 1).

**Table 1:** Distribution of cases and controls according to age * The p-value was calculated using the Mann-Whitney U test (for mean age) and the chi-square test (for age categories), and p<0.05 was statistically significant.

Age	Total (N = 467)	Cases (N = 232)	Controls (N = 235)	p-value*
18-25 years	69 (14.8)	28 (12.1)	41 (17.4)	0.116
26-30 years	219 (46.9)	112 (48.3)	107 (45.5)	
31-35 years	145 (31)	73 (31.5)	72 (30.6)	
>35 years	34 (7.3)	19 (8.2)	15 (6.4)	
Mean age (years)	27.09 ± 5.02	27.74 ± 5.15	26.43 ± 4.93	0.263

The difference in sociodemographic characteristics between cases and controls was comparable. A significantly higher proportion of cases had a history of pregnancy loss compared to controls (p = 0.042). Moreover, 68 (29.3%) cases and 48 (20.4%) controls reported that the current pregnancy was unplanned, and this difference was statistically significant (p = 0.026). A past history of depression was self-reported by 22.4% of pregnant women with GDM and 8.1% of pregnant women without GDM (p < 0.001) (Table 2).

**Table 2:** Distribution of cases and controls according to obstetric and psychological characteristics *The p-value was calculated using the chi-square test, and p<0.05 was statistically significant.

Characteristics	Total (N = 467)	Cases (N = 232)	Controls (N = 235)	p-value*
Gravida				
Primigravida	120 (25.7)	67 (28.9)	53 (22.6)	0.118
Multigravida	347 (74.3)	165 (71.1)	182 (77.4)	
History of pregnancy loss				
Yes	67 (14.3)	41 (17.7)	26 (11.1)	0.042*
No	400 (85.7)	191 (82.3)	209 (88.9)	
Complications in past pregnancy (n = 347)				
Yes	55 (15.9)	26 (15.8)	29 (15.9)	0.964
No	292 (84.1)	139 (84.2)	153 (84.1)	
Intent of current pregnancy				
Planned	351 (75.2)	164 (70.7)	187 (79.6)	0.026*
Unplanned	116 (24.8)	68 (29.3)	48 (20.4)	
History of depression				
Yes	71 (15.2)	52 (22.4)	19 (8.1)	<0.001*
No	396 (84.8)	180 (77.6)	216 (91.9)	

The mean EPDS score at 28 weeks of gestation was comparable between cases and controls. However, the mean EPDS score prior to delivery was significantly higher among women with GDM compared to those without GDM (Table 3). Eleven cases and three controls presented at a gestational age of <37 weeks, whereas 20 cases and 12 controls presented at ≥37 weeks with EPDS scores of 12-13. Similarly, one case presented at a gestational age of <37 weeks, whereas five cases and two controls presented at ≥ 37 weeks with EPDS ≥ 14.

**Table 3:** Distribution of cases and controls according to EPDS scores *The p-value was calculated using the Mann-Whitney U test (for mean EPDS score) and the chi-square test (for categorical data), and p<0.05 was statistically significant. EPDS: Edinburgh Postnatal Depression Scale, GA: gestational age, ANC: antenatal care.

Characteristics	Total (N = 467)	Cases (N = 232)	Controls (N = 235)	p-value*
EPDS score at 28 weeks GA				
≤8	424 (90.8)	206 (88.8)	218 (92.8)	0.138
9-11	43 (9.2)	26 (11.2)	17 (7.2)	
Mean EPDS score	5.44 ± 5.01	6.76 ± 5.21	5.11 ± 4.94	0.429
EPDS score at the last ANC visit prior to delivery				
EPDS ≤8	296 (63.4)	127 (54.7)	169 (71.9)	0.001*
<37 weeks	85 (18.2)	33 (14.2)	52 (21.8)	
≥37 weeks	211 (45.2)	94 (40.5)	117 (49.8)	
EPDS 9-11	117 (25.1)	68 (29.3)	49 (20.9)	
<37 weeks	44 (9.4)	24 (10.3)	20 (8.5)	
≥37 weeks	73 (15.6)	42 (18.1)	31 (13.2)	
EPDS 12-13	46 (9.9)	31 (13.4)	15 (6.4)	
<37 weeks	14 (3.0)	11 (4.7)	3 (1.3)	
≥37 weeks	32 (6.9)	20 (8.6)	12 (5.1)	
EPDS ≥ 14	8 (1.7)	6 (2.6)	2 (0.9)	
<37 weeks	1 (0.2)	1 (0.4)	0 (0)	
≥37 weeks	7 (1.5)	5 (2.1)	2 (0.9)	
Mean EPDS score	9.38 ± 6.47	10.30 ± 7.09	8.45 ± 5.87	<0.001*

A total of 15.9% (n = 37) of pregnant women with GDM and 7.2% (n = 17) of pregnant women without GDM had significant symptoms of antenatal depression, and this difference was statistically significant (p = 0.003) (Table 4).

**Table 4:** Distribution of cases and controls according to presence of significant symptoms of antenatal depression. *The p-value was calculated using the chi-square test, and p<0.05 was statistically significant. EPDS: Edinburgh Postnatal Depression Scale.

Significant symptoms of antenatal depression	Total (N = 467)	Cases (N = 232)	Controls (N = 235)	p-value*
Present (EPDS score ≥12)	54 (11.6)	37 (15.9)	17 (7.2)	0.003*
Absent (EPDS score <12)	413 (88.4)	195 (84.1)	218 (92.8)	

The sociodemographic and behavioural factors associated with significant symptoms of antenatal depression are depicted in Table 5. Pregnant women with symptoms of depression had a higher mean age compared to those without symptoms (p = 0.039). Those belonging to a lower socioeconomic group, having poor social support, and leading a sedentary lifestyle were associated with the presence of significant symptoms of antenatal depression. While a family history of diabetes was not associated with antenatal depression (p = 0.630), a family history of depression (p < 0.001) and a past history of depression (p < 0.001) were identified as being significantly associated with antenatal depression.

**Table 5:** Correlation of sociodemographic and behavioral factors with symptoms of antenatal depression * The p-value was calculated using the Mann-Whitney U test (for mean age) and the chi-square test (for categorical data), and p<0.05 was statistically significant.

Characteristics	Total (N = 467)	Depression (N = 54)	No depression (N = 413)	p-value*
Mean age (years)	27.09 ± 5.02	30.38 ± 7.21	26.65 ± 6.26	0.039*
Type of family				
Nuclear	279 (59.7)	29 (53.7)	250 (60.5)	0.336
Joint/extended	188 (40.3)	25 (46.3)	163 (39.5)	
Socioeconomic status				
Class I (upper)	40 (8.6)	1 (1.9)	39 (9.4)	0.038*
Class II (upper middle)	142 (30.4)	11 (20.4)	131 (31.7)	
Class III (lower middle)	146 (31.3)	23 (42.6)	123 (29.8)	
Class IV (upper lower)	139 (29.8)	19 (35.2)	120 (29.1)	
Perceived social support				
High	101 (21.6)	6 (11.1)	95 (23)	0.046*
Low	366 (78.4)	48 (88.9)	318 (77)	
Lifestyle				
Sedentary	147 (31.5)	40 (74.1)	107 (25.9)	<0.001*
Moderately active	320 (68.5)	14 (25.9)	306 (74.1)	
Gravida				
Primigravida	120 (25.7)	7 (13.0)	113 (27.4)	0.023*
Multigravida	347 (74.3)	47 (87.0)	300 (72.6)	
History of pregnancy loss				
Yes	67 (14.3)	16 (29.6)	51 (12.3)	0.001*
No	400 (85.7)	38 (70.4)	362 (87.7)	
Intent of current pregnancy				
Planned	351 (75.2)	33 (61.1)	318 (77.0)	0.011*
Unplanned	116 (24.8)	21 (38.9)	95 (23.0)	
Family history of diabetes				
Present	143 (30.6)	15 (27.8)	128 (31.0)	0.630
Absent	324 (69.4)	39 (72.2)	285 (69.0)	
Family history of depression				
Present	21 (4.5)	15 (27.8)	6 (1.5)	<0.001*
Absent	446 (95.5)	39 (72.2)	407 (98.5)	
Past history of depression				
Yes	71 (15.2)	41 (75.9)	30 (7.3)	<0.001*
No	396 (84.8)	13 (24.1)	383 (92.7)	
GDM in current pregnancy				
Yes	232 (49.7)	37 (68.5)	195 (47.2)	0.003*
No	235 (50.3)	17 (31.5)	218 (52.8)	

## Discussion

Depression during pregnancy commonly affects about 15.6% of women in developing countries, with a prevalence of around 12.8% during the second and third trimesters [18,19]. It has been associated with an increased risk of preterm birth, low birth weight, and caesarean deliveries [20]. The overall prevalence of significant depressive symptoms among our study participants was 11.6%. About 15.9% (n = 37) of pregnant women with gestational diabetes and 7.2% (n = 17) of women without gestational diabetes had significant symptoms of antenatal depression (p = 0.003). Furthermore, the mean EPDS score at the last antenatal visit prior to delivery was also significantly higher among the cases compared to controls (p < 0.001), demonstrating an association between depression and diabetes. Similar to these findings, it has been reported that the overall prevalence of depression was significantly higher in women with GDM (25.92%) than in those without GDM (10.38%), with the overall prevalence of antenatal depression being 18.32% [14]. In a large study of more than 11,000 women, Kozhimannil et al. reported that those with GDM during pregnancy were 1.85 times more likely to experience depression compared to women without GDM [21]. These investigators included women with type 1 diabetes, type 2 diabetes, and GDM in their sample. Based on their findings, healthcare providers may need to screen women with diabetes more frequently for depression during prenatal care visits. In addition, Arafa et al. reported in a previous study that GDM significantly increased the likelihood of postpartum depression [22]. A meta-analysis by Rotella et al. showed that depression is linked to a higher risk of developing diabetes in the general population (relative risk: 1.38, 95% confidence interval: 1.23-1.55) [23].

In the study by Byrn et al., the prevalence of antenatal depression was 20% among pregnant women with GDM and 13% among those without GDM [24]. They further observed that women with GDM were more likely to have a prior history of depression. In the present study as well, it was observed that a higher proportion of women with GDM had a history of depression. The clinical relevance of this finding lies in the need for earlier screening and more vigilant monitoring for GDM among women with a history of depression. Earlier research has proposed depression as a potential risk factor for the development of type 2 diabetes [25]. It has been suggested that depression-induced changes in neurotransmitter function may negatively affect glycaemic control by contributing to hyperglycaemia. Depression is associated with alterations in neurotransmitter function, particularly involving serotonin, norepinephrine, and dopamine. These neurochemical changes can disrupt hypothalamic-pituitary-adrenal axis regulation and increase sympathetic nervous system activity, leading to elevated cortisol and catecholamine levels. Such neuroendocrine disturbances promote insulin resistance, impair glucose metabolism, and may negatively affect glycaemic control. This may be the physiological mechanism through which depression predisposes a person to diabetes [26]. Additionally, studies have shown a significant association between depression and hyperglycaemia in individuals with type 1 and type 2 diabetes [27]. In a large study of 121260 women, Bowers et al. found that a prior history of depression was significantly associated with the development of GDM (odds ratio: 1.42, 95% confidence interval: 1.26-1.60) [28]. Our study reinforces the importance of recognising a history of depression as a significant risk factor for GDM.

Age plays a key role in pregnancy-related complications; in our study, women exhibiting significant antenatal depression were of higher age than those without depression (p = 0.039). A possible explanation may be that older pregnant women face greater cumulative stress and health challenges, which could increase their risk of depression [29]. Women with lower socioeconomic status and poorer perceived social support were more likely to experience antenatal depression (p < 0.05). Financial stress and limited resources in lower socioeconomic groups can contribute to mental health issues. The perception of social support is crucial; individuals who feel unsupported are more prone to depression, regardless of their actual family structure [30]. Chalise et al. also identified low perceived social support as an important determinant of antenatal depression [31]. It has been reported that depression was more prevalent among women with higher levels of education [14]. A significantly higher proportion of cases had a history of pregnancy loss (17.7% vs. 11.1%; p = 0.042) and unplanned pregnancies (29.3% vs. 20.4%; p = 0.026). These factors were also associated with an increased risk of antenatal depression. Pregnancy loss and unplanned pregnancies can lead to increased stress, anxiety, and feelings of unpreparedness, contributing to the development of GDM and antenatal depression. The emotional impact of past pregnancy loss can heighten concerns during subsequent pregnancies, increasing the risk of depression [32,33]. Our observations are in line with the findings of the study by Chalise et al. [31]. It has been reported that women with unplanned pregnancies have a three-fold increase in the odds of depression (AOR: 3.43, 95% CI: 1.78 to 6.62) compared to women with planned pregnancies [34]. Multigravida not only have greater exposure to potential complications associated with pregnancy and childbirth, but they also experience increased stress as they must meet the demands of a newborn while continuing to care for their other children [35]. This could be a contributing factor to the higher levels of psychological distress seen in multigravida relative to primigravida.

The strengths of this study include systematic data collection using a standardised protocol, enrolment of participants through consecutive sampling, and the use of validated diagnostic and screening tools. GDM was diagnosed using standard DIPSI criteria, and depressive symptoms were assessed using the EPDS, a well-validated instrument for antenatal populations. These methodological strengths enhance the reliability, internal validity, and reproducibility of the findings. Despite these strengths, the study has several limitations. Although the sample size was adequate for statistical analysis, it may not be sufficient to generalise the findings to all pregnant women, particularly those from different geographical or socioeconomic backgrounds. Furthermore, because the study was conducted at a single hospital, the results may not be fully representative of the wider population and therefore require validation in larger, multicentre studies. The study relied on self-reported data for variables such as history of depression, family history, lifestyle habits, and perceived social support. Since several key variables were self-reported, the findings may be affected by recall and social desirability biases, potentially reducing the precision of the information provided. Owing to the case-control design, causal relationships between GDM and antenatal depression could not be established. Moreover, due to limited resources, the participants could not be followed beyond delivery, and therefore, the development of postpartum depression, especially in women who had antenatal depression or gestational diabetes, could not be evaluated. While this study provides important insights into the association between GDM and antenatal depression, addressing these limitations in future research will enhance the understanding and management of these conditions. Conducting larger, longitudinal, and multicentre studies with comprehensive data collection methods will help develop targeted interventions to support the mental health and overall well-being of pregnant women.

## Conclusions

The findings of the study highlight the need for comprehensive antenatal care that includes routine mental health screening and appropriate support for depression, especially for women with GDM. Factors such as maternal age, socioeconomic status, perceived social support, pregnancy history, and previous mental health issues should be carefully considered when managing and supporting pregnant women. Addressing these factors is essential for the prevention and effective treatment of antenatal depression. Interventions such as regular mental health assessment, lifestyle modification support, counselling, and a stronger social support system can play an important role in reducing risks and improving the overall well-being of pregnant women.
